# *Cellu*lite and extracorporeal *Shock*wave therapy (CelluShock-2009) - a Randomized Trial

**DOI:** 10.1186/1472-6874-10-29

**Published:** 2010-10-26

**Authors:** Karsten Knobloch, Beatrice Joest, Peter M Vogt

**Affiliations:** 1 Plastic, Hand and Reconstructive Surgery, Hannover Medical School, Germany

## Abstract

**Background:**

Cellulite is a widespread problem involving females' buttocks and thighs based on the female specific anatomy. Given the higher number of fat cells stored in female fatty tissue in contrast to males, and the aging process of connective tissue leads to an imbalance between lipogenesis and lipolysis with subsequent large fat cells bulging the skin. In addition, microcirculatory changes have been suggested, however remain largely unknown in a controlled clinical setting. We hypothesize that the combination of extracorporeal shockwave and a daily gluteal muscle strength program is superior to the gluteal muscle strength program alone in cellulite.

**Methods/Design:**

Study design: Randomized-controlled trial. IRB approval was granted at Hannover Medical School, Germany on May 22, 2009. For allocation of participants, a 1:1 ratio randomization was performed using opaque envelopes for the concealment of allocation. Reporting: according to CONSORT 2010. Eligible patients were females aged 18 or over and 65 or younger with cellulite with documented cellulite 1°-4° according to the Nürnberger score. Exclusion criteria were suspected or evident pregnancy, no cellulite, no informed consent or age under 18 years or above 65 years. Patients were recruited by advertisements in local regional newspapers and via the Internet. Analysis: Intention-to-treat. Outcome parameters: a) Photonumeric severity scale, b) Nürnberger Score, c) circumference measurements, d) capillary blood flow, e) tissue oxygen saturation, f) postcapillary venous blood flow. Intervention group: Six sessions of extracorporeal focused shock wave for six sessions (2000 impulses, 0,25 mJ/m2 every 1-2 weeks) at both gluteal and thigh regions plus a specific gluteal strength exercise training. Control group: Six sessions of sham extracorporeal focused shock wave for six sessions (2000 impulses, 0,01 mJ/m2 every 1-2 weeks) at both gluteal and thigh regions plus a specific gluteal strength exercise training. Follow-up: 12 weeks. Blinding was achieved for all participants enrolled in the trial, the photograph taking the digital images for the primary outcome measure, the two assessors of the outcome measures, all additional health care providers and for the analyst from the biometrical department. Only one researcher (BJ) was aware of the group assignment performing the randomisation and the extracorporeal shock wave therapy.

**Discussion:**

This randomised-controlled trial will provide much needed evidence on the clinical effectiveness of focused extracorporal shock wave therapy as an adjunct to gluteal strength training in females suffering cellulite.

**ClinicalTrials.gov identifier:**

NCT00947414

## Background

Given the fact that publishing study protocols might improve registration, reporting as well as recruitment [1], we present our randomized-controlled trial entitled "CelluShock-2009 (ClinicalTrials.gov NCT00947414) in the following.

Cellulite is a widespread problem involving females' buttocks and thighs based on the female specific anatomy. Given the higher number of fat cells stored in female fatty tissue in contrast to males, and the aging process of connective tissue leads to an imbalance between lipogenesis and lipolysis with subsequent large fat cells bulging the skin. In addition, microcirculatory changes have been suggested, however remain largely unknown in a controlled clinical setting.

Non-randomized clinical data suggest that extracorporal shock wave therapy applied as acoustic wave therapy is beneficial in terms of improved skin elasticity and revitalizing dermis in cellulite [2,3].

The following non-controlled studies examined the effect of extracorporeal shock wave therapy on cellulite with various outcome measures (Table 1).

**Table 1:** Non-randomised clinical studies on the effect of extracorporeal shock wave therapy on cellulite with different outcome measures applied.

Author	Journal	Year	Patient number	Outcome	Design
Angehrn F [4]	Clin Interv Aging	2007	21	HR-Ultrasound	Case series
Kuhn C [5]	Clin Interv Aging	2008	1	HR Ultrasound, histopathology	Case Study
Christ C [6]	Aesthetic surgery journal	2008	59	Dermascan US DermaLab System	Cohort study

Recently, a small size (n = 25) randomised-controlled trial with large confidence intervals has been published [7]. Six sessions over four weeks using the D-ACTOR^® ^200 by Storz Medical improved depressions, elevations, roughness, and elasticity within three months. However, to date we do not have any evidence regarding the effect of gluteal home-based strength training with or without extracorporeal shockwave therapy on the clinical outcome in cellulite in terms of digital images, microcirculation and patient-self-reported assessment.

### Hypothesis

The combination of extracorporeal shockwave and a daily gluteal muscle strength program is superior to the gluteal muscle strength program alone in cellulite.

## Methods/Design

The study protocol is composed according to the most recent CONSORT 2010 recommendations for transparent reporting of randomised-controlled trials http://www.consort-statement.org[8]. While the group stressed, that "*CONSORT does not include recommendations for designing, conducting, and analyzing trials*" [9] we believe that authors will thoroughly benefit from the very beginning of planning RCTs from the consideration of the items addressed in the recommendations.

### Ethics and Registration

This RCT was approved on May 22, 2009 at the ethics (IRB) at Hannover Medical School, Germany under the German title "Stosswellentherapie und Krafttraining zur Therapie der Cellulite - eine randomsiert-kontrollierte Studie" (Nr. 5206). The study is registered at ClinicalTrials.gov with ClinicalTrials.gov identifier: NCT00947414.

### Design of the study

#### (CONSORT Item 3a)

This is a single-center superiority randomised-controlled trial with a 1:1 parallel group randomisation

### Setting of the study

#### (CONSORT Item 4b)

This study took place at the Hannover Medical School, Germany in the department for Plastic, Hand and Reconstructive Surgery starting in June 2009. Hannover Medical School is a University Hospital with 7040 employees, among them 1221 physicians and 2839 medical students. In 2009, 1444 clinical beds achieved 54628 stationary cases with a 90% bed occupancy rate. This trial is a single center randomised-controlled trial.

Hannover had on June 30, 2009 a population of 519212 people with 272541 females accounting for 51.8%. 77,378 people were aged below 18 (14,7%), 128,855 60 years or older (24.5%). Uncontrolled prevalence report estimate up to 85% of females suffering from cellulite of various degrees. This elaboration provides some further information whether the settings and locations used in this study are relevant for another given avid reader in terms of external validity, since the results obtained here do not necessarily correlate to a different environment [10].

### Participants

#### (CONSORT Item 4a)

Eligible patients were females aged 18 or over and 65 or younger with cellulite who met the eligibility criteria with documented cellulite 1°-4° according to the Nürnberger score. Exclusion criteria were suspected or evident pregnancy, no cellulite, no informed consent or age under 18 years or above 65 years. Patients were recruited by advertisements in local regional newspapers and via the Internet. Some patients from the United States used the http://www.ClinicalTrials.gov reference to approach and seek for participation. Given the broad inclusion criteria in terms of age (18-65 years) and degree of cellulite (Nünberger score 1°-4°) we thought to improve external validity, which is generalisability [11]. Furthermore, in terms of gender, female participants are the minority of randomized-controlled trials per se. An analysis of published RCTs from 1994 to 2006 revealed that patients were excluded for age in 72% and for female gender in 39% [12], which limits external validity of a given RCT. Both, pre- and postmenopausal women are included with documentation of their status and concomitant medication.

### Interventions

#### (CONSORT Item 5)

**Figure 1:**
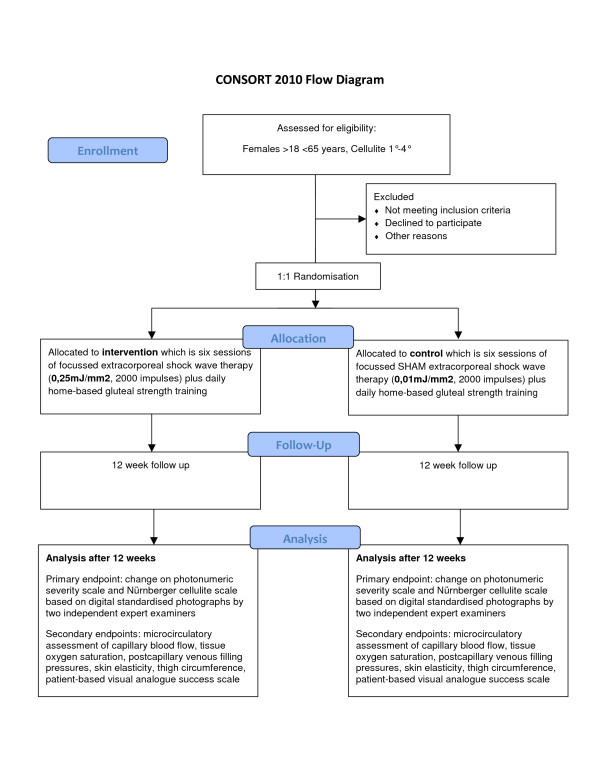
**CONSORT patient flow chart**.

In CelluShock-2009 patients were randomly assigned with a 1:1 ratio to either extracorporeal shock-wave therapy with a 25-fold higher energy than the sham extracorporeal shock wave therapy. Both groups additionally participated in a home-based daily gluteal thigh exercise program (Figure 1 CONSORT flow chart).

• Intervention group:

◦ Six sessions of extracorporeal shockwave therapy (every 1-2 weeks) with focussed shock waves (2000 impulses, **0, 25 mJ/mm2**) plus daily home-based gluteal strength exercise

• Control group:

◦ Six sessions of sham extracorporeal shock wave (2000 impulses, **0, 01 mJ/mm2**, every 1-2 weeks) plus daily home-based gluteal strength exercise

Extracorporeal shock wave therapy was applied using a STORZ focussed Duolith machine as acoustic wave treatment. Acoustic wave are sound wave characterised by high pressure in comparison to ambient pressure. The generation of sound waves used for medical application are generated extracorporeal, thus extracorporeal acoustic wave therapy or shock wave therapy. As acoustic waves propagate according to the laws of acusto-optics, any change in the acoustic properties might cause an impedance jump at the border of tissues. Energy will thus be released in the target tissue, such as lymphangion contraction and improved permeability of cellular membranes in terms of acoustic wave therapy.

In order to increase the motivation of the participating females we added a daily home-based gluteal strength exercise program. Twice a day (in the morning and the evening) two different exercises were performed with 15 repetitions for each leg (Figures 1a/b, 2a/b).

**Figure 2:**
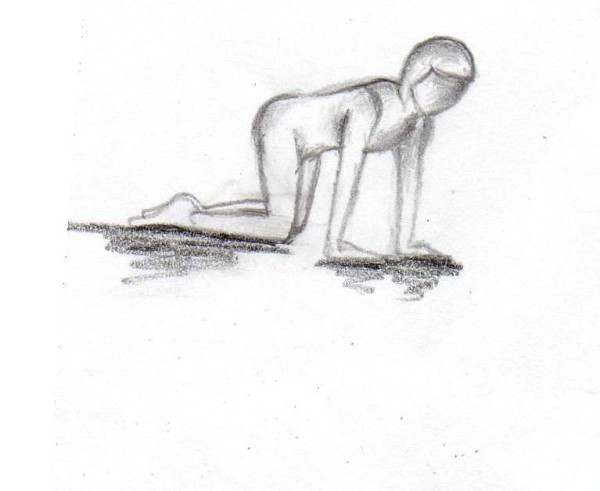
**First exercise (15 repetitions per leg twice a day over 12 weeks)**.

The daily workup was noted in a exercise log to improve participants' compliance.

This elaboration was that detailed in order to provide the reader with sufficient detail to replicate the intervention in a similar fashion [13] and to meet the CONSORT recommendations for reporting of randomised trials of non-pharmacologic treatment [14].

### Primary and secondary outcome measures

#### (CONSORT Item 6a)

The **primary endpoint **with respect to efficacy of the combined extracorporeal shock wave therapy and gluteal strength exercises vs. sham extracorporeal shock wave therapy and the same gluteal strength exercise program was the change on digital photographs.

Given the recent publication of a photonumeric severity score [15] providing reliable, comprehensive, and reproducible severities from 0 to 15 we added this tool in 2009 during the recruiting phase in contrast to the initially proposed Nürnberger and Müller score documented in the ClinicalTrials.gov file. This 4-graded scale will be additionally determined by two independent blinded assessors.

### 4-graded scale according to Nürnberger and Müller [16]

• Stage 0: No dimpling when the skin is pinched

• Stage I: No spontaneous displays of alterations, pressure is required to show dimpling

• Stage II: Dimpling is visible when standing, not while lying

• Stage III: Skin alterations while both standing and lying

The classification was performed based on standardized photographs taken by a professional medical photographer at baseline and after 12 weeks after randomisation in both groups. The assessment of the anonymous digital images was carried out by two blinded assessors who were not aware of either the study arm or the fact that it is a baseline or a follow-up photograph taken 12 weeks after study initiation.

In order to overcome the problems of interpretation associated with multiplicity of analyses were decided to choose the aforementioned clinical endpoint which is a visual one as the primary endpoint and apparatively derived results as secondary endpoints in CelluShock.

**Secondary endpoints **of CelluShock RCT were:

• Change of circumference of the thigh [cm]

• Microcirculatory change of capillary blood flow of the thigh using combined Laser-Doppler & spectrophotometry (Oxygen-to-see system, LEA Medizintechnik, Germany, http://www.lea.de)

• Microcirculatory change of tissue oxygen saturation of the thigh using combined Laser-Doppler & spectrophotometry (Oxygen-to-see system, LEA Medizintechnik, Germany, http://www.lea.de)

• Microcirculatory change of postcapillary venous filling pressure of the thigh using combined Laser-Doppler & spectrophotometry (Oxygen-to-see system, LEA Medizintechnik, Germany, http://www.lea.de)

• Skin elasticity using the Cutometer^® ^(Cutometer MPA 580, Kosmetik Konzept KOKO GmbH & Co KG, Leichlingen, http://www.dermaviduals.de) [17-20]

• Self-assessment of the success on a visual analogue scale 0-10 (0 = no change, 10 = fully satisfied)

The combined laser Doppler flowmetry & spectrophotometry system Oxygen-to-see (LEA Medizintechnik, http://www.lea.de) is a non-invasive real-time measure to determine three distinct parameters of tissue microcirculation simultaneously in 2 and 8 mm tissue depths [21-26]:

• Capillary blood flow

• Tissue oxygen saturation

• Postcapillary venous filling pressures

All patients were measured at baseline and after 12 weeks regarding the primary and all secondary endpoints. Microcirculatory assessment was performed as microcirculatory gluteal mapping on various standardized locations (10 on each thigh) in a prone position.

### Change of trial outcomes

#### (CONSORT Item 6b)

To date, during the recruiting phase of CelluShock there has been no change in any primary or secondary outcome nor is it intended to change it.

### Power calculation

#### (CONSORT Item7a)

To detect at least a change of one class in the four-graded Nürnberger scale of cellulite with a two-sided 5% significance with a 80% power a sample size of 50 participants with an estimated drop-out rate of 15% was calculated.

### Interim analysis

#### (CONSORT Item 7b)

Due to the calculated rather short recruiting phase of only 2 years and the 12-week follow-up, we do not plan to perform an interims analysis. Furthermore, due to the low if at all adverse effects reported for extracorporeal shock wave therapy applied in our dosages we do not expect that the trial has to be ended or stopped early for ethical reasons.

### Randomization

#### (CONSORT Item 8a)

For allocation of participants, a 1:1 ratio randomization was performed using opaque envelopes for the concealment of allocation. In order to minimize the chance of bias we used [27]

a) Opaque, sealed and serial numbered envelopes

b) opened sequentially and only after the participant's name and further details were written on the envelope

c) kept them in a locked and secure place.

#### (CONSORT Item 8b)

Participants were randomly assigned following simple 1:1 randomisation procedures using opaque and sealed envelopes to one of the two study arms (interventional or control arm) without any restriction.

#### (CONSORT Item 9)

The allocation sequence was concealed from the researcher (BJ) enrolling and assessing participants in sequentially numbered, opaque, sealed envelopes [28].

### Allocation sequence

#### (CONSORT Item 10)

Sequentially numbered, opaque and sealed envelopes were opened by one researcher (BJ) after the participants was deemed appropriate fulfilling all inclusion criteria and no exclusion criteria as stated above.

### Blinding

#### (CONSORT Item 11a)

Blinding was achieved for all participants enrolled in the trial, the photograph taking the digital images for the primary outcome measure, the two assessors of the outcome measures, all additional health care providers and for the analyst from the biometrical department. Only one researcher (BJ) was aware of the group assignment performing the randomisation and the extracorporeal shock wave therapy. As stated above, the extracorporeal shock wave therapy was applied in both groups (intervention and control group) with an identical number of impulses (2000 per thigh) and the same frequency (4 Hz), thus the time of the procedure was identical. Only the energy of either 0,25 mJ/mm2 or 0,01 mJ/mm2 applied was different between both groups, which were hidden from the patient displayed on the STORZ Duolith console. Thus, only the operator of the shock wave console (BJ) was aware which energy to chose.

The assessment of the primary and secondary outcomes was performed by blinded assessors independently from each other without any clue whether the digital image displayed was before or after therapy or with group (intervention or control group) was randomised. In addition, no patient was aware whether he was in the intervention or the control group, since all underwent similar shock wave therapy over the same period, however, with a 25-fold delta in energy.

### Similarities of interventions

#### (CONSORT Item 11b)

As stated above, the extracorporeal shock wave therapy was applied in both groups (intervention and control group) with an identical number of impulses (2000 per thigh) and the same frequency (4 Hz), thus the time of the procedure was identical. Only the energy of either 0,25 mJ/mm2 or 0,01 mJ/mm2 applied was different between both groups, which were hidden from the patient displayed on the STORZ Duolith console. Thus, only the operator of the shock wave console (BJ) was aware which energy to chose.

Compliance to the extracorporeal shock wave therapy was naturally measured. In terms of compliance to the daily suggested gluteal thigh strength exercises, a daily log was recorded over the 12 weeks in addition to serial interviews with the participants throughout the trial addressing any problems in compliance with the strength program.

As the CONSORT 2010 statement does not include any longer the suggestion to obtain information what the participants randomised thought which group they were at the end of the trial, we do not record this item [29].

### Statistics

#### (CONSORT Item 12a)

The primary endpoint was change of Nürnberger scale assessed on digital standardised photographs by two independent expert examiners. An intention-to-treat analysis was applied that once randomised the patients is retained in the allocated group (intervention group or control group) for analysis whatever occurs. This is to limit bias in this superiority RCT.

### CONSORT flow chart

**Figure 3:**
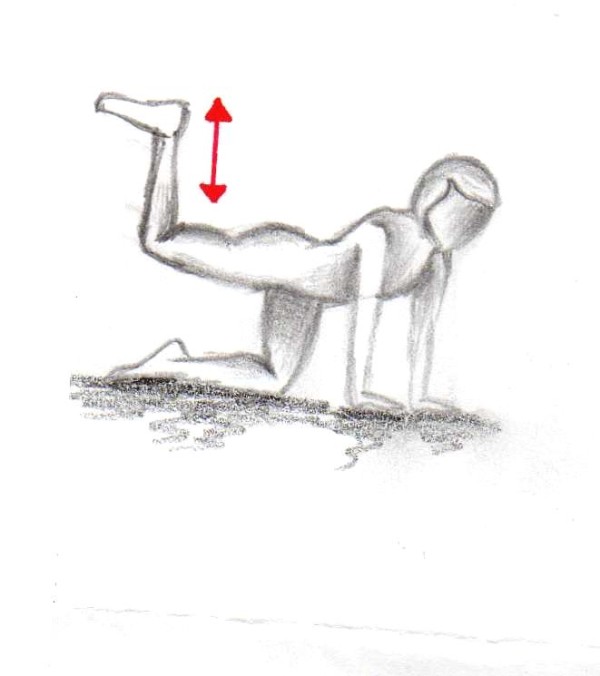
**First exercise (15 repetitions per leg twice a day over 12 weeks)**.

Figure 3 highlight the proposed patient flow throughout the CelluShock-2009 randomised trial (Figure 3).

## Discussion

Given the aforementioned clinical trials applying extracorporeal shockwave therapy in females suffering from cellulite we seek to evaluate in a large randomised-controlled trial the effects of strength training and the additional effect of shockwave therapy on the degree of cellulite. While to date only one small size randomised controlled trial [7] with wide confidence intervals has been published, we try to overcome the methodological shortcomings of previous trials in CelluShock-2009. To date, there is no information whether gluteal strength training is effective in a clinical trial in cellulite, which we will elaborate on. Furthermore, the superiority of additional extracorporeal shockwave therapy in addition to daily gluteal strength training in females suffering from cellulite will be the research question to be answered by this RCT.

## Competing interests

No one of the authors has any personal or financial relationship with other people or organizations involved or targeted by this RCT. No author has any competing interests'.

## Authors' contributions

**KK **is the principal investigator (PI) of this RCT at Hannover Medical School, Plastic, Reconstructive Surgery, Germany, involved in the conception and design, acquisition of data, blinded analysis of the data, interpretation of the data and writing this manuscript. **BJ **is involved in the conception and design, acquisition of data, interpretation of data and writing this manuscript. **PMV **as head of department of Plastic, Hand and Reconstructive Surgery, Hannover Medical School, Germany is actively involved in the conception and design, acquisition of data, blinded analysis of the data, interpretation of the data and writing this manuscript. All authors gave their final approval of this version to be published.

**Figure 4:**
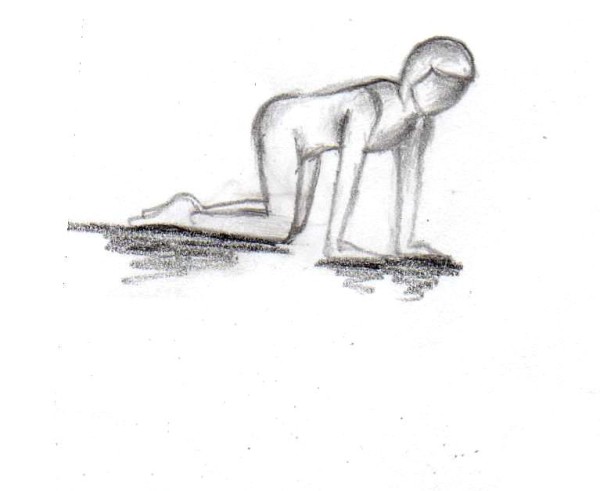
**Second exercise (15 repetitions per leg twice a day over 12 weeks)**.

**Figure 5:**
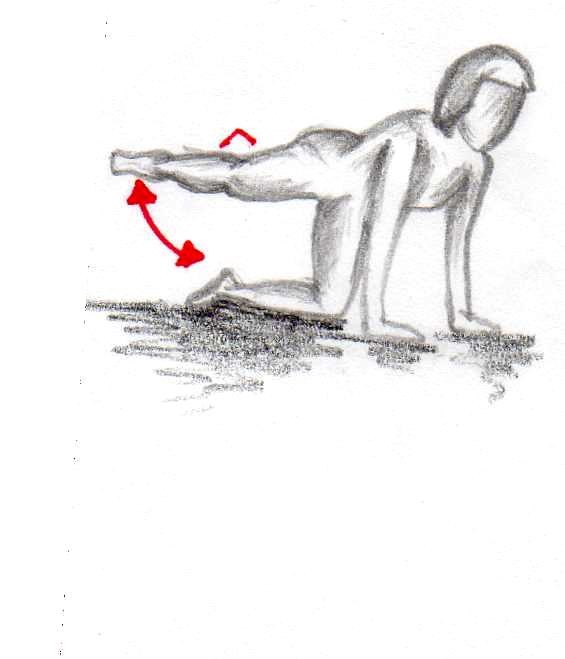
**Second exercise (15 repetitions per leg twice a day over 12 weeks)**.

## Pre-publication history

The pre-publication history for this paper can be accessed here:

http://www.biomedcentral.com/1472-6874/10/29/prepub

## References

[B1] Godlee F Publishing study protocols: making them visible will improve registration, reporting and recruitment BMC News and Views 2001 2 4

[B2] Christ C Brenke G Sattler G Steigerung der Hautelastizität und Revitalisierung der Dermis bei Cellulite und Bindegewebsschwäche durch die extrakorpoale Acoustic Wave Therapy (AWT) Ästhetische Dermatologie 2008 1 2 10

[B3] Sattler G Pohl U Raegener K Pilotstudie akustische Wellentherapie (AWT) bei Cellulite Äathetische Dermatologie 2008 2 16 25

[B4] Angehrn F Kuhn C Voss A Can cellulite be treated with low-energy extracorporal shock wave therapy? Clin Interv Aging 2007 2 4 623 30 18225463 10.2147/cia.s1721 PMC2686339

[B5] Kuhn C Angehrn F Sonnabend O Voss A Impact of extracorporal shock waves on the human skin with cellulite: a case study of an unique instance Clin Interv Aging 2008 3 1 201 10 18488890 10.2147/cia.s2334 PMC2544366

[B6] Christ C Brenke R Sattler G Siems W Novak P Daser A Improvement in skin elasticity in the treatment of cellulite and connective tissue wekness by means of extracorporal pulse activation therapy Aesthet Surg 2008 28 5 538 44 10.1016/j.asj.2008.07.011 19083577

[B7] Adatto M Adatto-Neilson R Servant JJ Vester J Novak P Krotz A Controlled, randomized study evaluating the effects of treating cellulite with AWT^®^/EPAT^®^ J Cosmet Laser Ther 2010 12 176 182 10.3109/14764172.2010.500392 20590369

[B8] Schulz KF Altman DG Moher D for the CONSORT group CONSORT 2010 statement: updated guidelines for reporting parallel group randomised trials BMC Medicine 2010 8 18 10.1186/1741-7015-8-18 20334633 PMC2860339

[B9] Schulz KF Altman DG Moher D Fergusson D CONSORT 2010 changes and testing blindness in RCTs Lancet 2010 375 9721 1144 6 10.1016/S0140-6736(10)60413-8 20338625

[B10] Weiss NS Koepsell TD Psaty BM Generalizability of the results of randomized trials Arch Intern Med 2008 168 133 5 10.1001/archinternmed.2007.30 18227357

[B11] Rothwell PM External validity of ranodmised controlled trials: „to whom do the results of this trial apply?" Lancet 2005 365 82 93 10.1016/S0140-6736(04)17670-8 15639683

[B12] Van Spall HG Toren A Kiss A Fowler RA Eligibility criteria of randomized controlled trials published in high-impact general medical journals: a systematic sampling review JAMA 2007 297 11 1233 40 10.1001/jama.297.11.1233 17374817

[B13] Glasziou P Meats E Heneghan C Shepperd S What is missing from descriptions of treatment in trials and reviews? BMJ 2008 336 1472 4 10.1136/bmj.39590.732037.47 18583680 PMC2440840

[B14] Boutron I Moher D Altman DG Schulz KF Ravaud P Extending the CONSORT statement to randomized trials of nonpharmacologic treatment: explanation and elaboration Ann Intern Med 2008 148 295 309 18283207 10.7326/0003-4819-148-4-200802190-00008

[B15] Hexsel DM Dal'Forno TD Hexsel CL A validated photonumeric cellulite severity scale J Eur Acad Dermatol Venereol 2009 23 5 523 8 10.1111/j.1468-3083.2009.03101.x 19220646

[B16] Nürnberger F Müller G So-called cellulite: An invented disease J Dermatol Surg Oncol 1978 4 221 9 632386 10.1111/j.1524-4725.1978.tb00416.x

[B17] Lautenschläger H Skin diagnosis - based on measuring results Kosmetik International 2008 10 54 56

[B18] Lautenschläger H Skin analysis - customer interview to complement measurements Kosmetik International 2004 8 72 4

[B19] Lautenschläger H Skin analysis - with the support of modern instruments Kosmetik International 2003 3 102 4

[B20] Lautenschläger H Instruments for skin analysis Kostmetik International 2001 1 90

[B21] Knobloch K Lichtenberg A Pichlmaier M Tomaszek S Krug A Haverich A Palmar microcirculation after harvesting of the radial artery in coronary revascularisation Ann Thorac Surg 2005 79 3 1026 30 10.1016/j.athoracsur.2004.03.026 15734429

[B22] Knobloch K Kraemer R Lichtenberg A Jagodzinski M Gossling T Richter M Zeichen J Hufner T Krettek C Achilles tendon and paratendon microcirculation in midportion and insertional tendinopathy in athletes Am J Sports Med 2006 34 1 92 7 10.1177/0363546505278705 16219947

[B23] Reenalda J Van Geffen P Nederhand M Jannink M Ijzerman M Rietman H Analysis of healthy sitting behaviour: interface pressure distribution and subcutaneous tissue oxygenation J Rehabil Res Dev 2009 46 5 577 86 10.1682/JRRD.2008.12.0164 19882492

[B24] Knobloch K Lichtenberg A Pichlmaier M Mertsching H Krug A Klima U Haverich A Microcirculation of the sternum following harvesting of the left internal mammary artery Thorac Cardiovasc Surg 2003 51 5 255 9 10.1055/s-2003-43083 14571341

[B25] Kraemer R Lorenzen J Rotter R Vogt PM Knobloch K Achilles tendon suture deteriorates tendon capillary blood flow with sustained tissue oxygen saturation - an animal study J Orthop Surg Res 2009 4 32 10.1186/1749-799X-4-32 19674439 PMC2731078

[B26] Knobloch K Grasemann R Spies M Vogt PM Midportion Achilles tendon microcirculation after intermittent combined cryotherapy and compression compared with cryotherapy alone: a randomized trial Am J Sports Med 2008 36 11 2128 38 10.1177/0363546508319313 18641371

[B27] Altman DG Schulz KF Statistics notes: concealing treatment allocation in randomised trials BMJ 2001 323 446 7 10.1136/bmj.323.7310.446 11520850 PMC1121039

[B28] Schulz KF Chalmers I Grimes DA Altman DG Assessing the quality of randomization from reports of controlled trials published in obstetrics and gynecology journals JAMA 1994 272 125 8 10.1001/jama.272.2.125 8015122

[B29] Sackett DL Turning a blind eye: why we don't test for blindness at the end of our trials BMJ 2004 328 1136 10.1136/bmj.328.7448.1136-a 15130997 PMC406365

